# Enlarged perivascular spaces are linked to freezing of gait in Parkinson's disease

**DOI:** 10.3389/fneur.2022.985294

**Published:** 2022-08-19

**Authors:** Fangju Lin, Baoling Yang, Ying Chen, Wei Zhao, Binghan Li, Weihua Jia

**Affiliations:** Department of Neurology, Beijing Shijingshan Hospital, Shijingshan Teaching Hospital of Capital Medical University, Beijing, China

**Keywords:** Parkinson's disease, freezing of gait, perivascular space, centrum semiovale, basal ganglia, cerebral small vessel diseases

## Abstract

**Objective:**

Freezing of gait (FOG) is one of common and disabling gait impairments of Parkinson's disease (PD). White matter hyperintensity (WMH) and lacunes, as common manifestations of cerebral small vessel diseases (CSVD), have been reported to be associated with gait function in PD patients. However, in the cases with FOG which present with extensive WMH or lacunes, it actually is difficult to distinguish pure PD pathology from vascular origin or combined effects. So far little is known about the correlation between enlarged perivascular space (PVS) and FOG in PD patients. This study aims to explore the role of enlarged PVS in FOG in PD patients.

**Methods:**

A total of 95 patients with PD in the absence of obvious WMH and lacunes were included in our study, which were divided into PD-FOG (+) group and PD-FOG (-) group. Demographic and clinical data were investigated. Enlarged PVS in the centrum semiovale (CSO) and basal ganglia (BG) were assessed. The association between enlarged PVS and FOG in patients with PD was analyzed using the multivariate models and the Spearman's correlation.

**Results:**

There were 36 PD patients grouped into PD-FOG (+) (37.9%), with an older age, a longer PD disease duration, and larger numbers of enlarged PVS in CSO and BG compared with PD-FOG (-) group. The highest-severity degree of enlarged PVS burden in CSO was independently associated with FOG in patients with PD [adjusted odds ratio (OR), 3.869; *p* = 0.022 in multivariable model]. The percentages of FOG case increased accompanied by the aggravation of enlarged PVS located in CSO. The grade and count of enlarged PVS in CSO and BG both correlated with FOGQ score in PD patients.

**Conclusion:**

Enlarged PVS, particularly in CSO, are associated with FOG in patients with PD, which provides a novel perspective for the mechanisms of FOG in PD.

## Introduction

Freezing of gait (FOG) is one of the most common and disabling gait impairments of Parkinson's disease (PD). FOG is characterized by sudden and transient episodes of inability to generate effective forward stepping typically triggered by gait initiation, turning, traversing narrow passages, or dual task (1). It is generally accepted that neural pathways mainly involving the striatum, motor cortex and pedunculopontine nucleus exert critical roles in locomotion of gait. Impairments in sensation processing and integration, executive cognition, psychological and emotional problems are also thought to associate with the pathophysiology of FOG in patients with PD (2, 3). However, mechanisms underlying FOG are still not completely elucidated.

Cerebral small vessel diseases (CSVD), especially white matter hyperintensity (WMH) as the most common neuroimaging biomarker of CSVD, have been intensively reported to plausibly associate with motor and gait function in PD patients (4, 5). However, a number of definite PD patients with gait impairments such as FOG present with only mild WMH or relatively clean imaging of brain in clinical scenarios. Furthermore, a clinicopathological study found a lower prevalence of vascular risk factors and CSVD pathology in autopsy-proven PD patients compared with community-dwelling controls (6). Moreover, it actually is difficult to distinguish pure PD pathology from cerebrovascular origin or combined effects which should be identified as the culprit of gait function when PD patients present with obvious WMH or lacunes. In view of what mentioned above, the relationship between CSVD and gait in PD patients still seems to be complicated and confusing.

Perivascular spaces (PVSs), also called as Virchow-Robin spaces, are interstitial fluid-filled spaces surrounding the perforating brain blood vessels, which are functionally involved in both interstitial fluid and glymphatic drainage systems (7, 8). PVSs are microscopic but visible on MRI when enlarged. Due to anatomical and physiological features, enlarged PVS is considered quite distinct from other CSVD imaging manifestations (WMH, lacune, and microbleed), although it is classified as one of markers of CSVD (9, 10). Enlarged PVSs have been reported to be associated with aging, vascular diseases, and neurodegenerative diseases (11–13). However, little is known about the correlation between enlarged PVSs and FOG in PD patients. It is clinically found that enlarged PVSs could be observed in PD patients with FOG, whereas other CSVD imaging markers such as WMH are absent, which thus come into our notice. There has been evidence showing that normal-sized PVS burden was correlated with the progression to FOG in PD (14). Also, enlarged PVS might reflect the poor interstitial fluid drainage of α-synuclein which could lead to gait dysfunction of PD (11). Therefore, we hypothesized that enlarged PVS may be associated with FOG of PD. The objective of this study was to explore the role of enlarged PVSs in FOG in PD patients in the absence of extensive WMH and lacunes.

## Materials and methods

### Study design and patients

This was a cross-sectional and single-center study. We consecutively recruited idiopathic patients with PD from Department of Neurology in Beijing Shijingshan Hospital from January 2020 to December 2021. The inclusion criteria were as follows: (1) age ≥55 years; (2) patients with the diagnosis of clinical definite PD according to the Movement Disorder Society (MDS) Clinical Diagnostic Criteria (15); (3) patients without contraindications to Magnetic Resonance Imaging (MRI) scan and underwent MRI scan of brain. The exclusion criteria were as follows: (1) evidence of secondary, atypical, or hereditary parkinsonism; (2) history of stroke, head trauma or tumor; (3) an inability to cooperate or communicate, such as severe dementia or psychiatric disorders; (4) brain MRI showing obvious lesions including WMH (Fazekas grade ≥2), lacunes (any lacunes located in BG or ≥3 lacunes in other regions), microbleeds, hydrocephalus, tumors, et al.; (5) patients with gait problems that could not exclude the possibility of being secondary to orthopedic issues, visual impairments, ataxia, et al.

### Clinical assessment of PD

The socio-demographic information of patients, including age, gender, years of education, past medical history, history of smoking and alcohol use, were recorded. The severity and stage of PD was evaluated using MDS Unified Parkinson**'**s Disease Rating Scale Part III (MDS-UPDRS III) and Hoehn and Yahr (HY) staging. FOG was assessed with FOG questionnaire (FOGQ) (16) combined with history and examination by two experienced neurologists. FOG was defined as a score of one or more on item 3 of FOGQ (feeling like feet being glued to the floor) and the neurologist would explain and demonstrate freezing if required. Based on this, patients were divided into two groups, PD-FOG (+) and PD-FOG (-). Cognitive function was assessed using the Mini-Mental State Examination (MMSE) and Montreal Cognitive Assessment (MoCA), which were adjusted for years of education.

### Brain MRI acquisition

**Figure 1:**
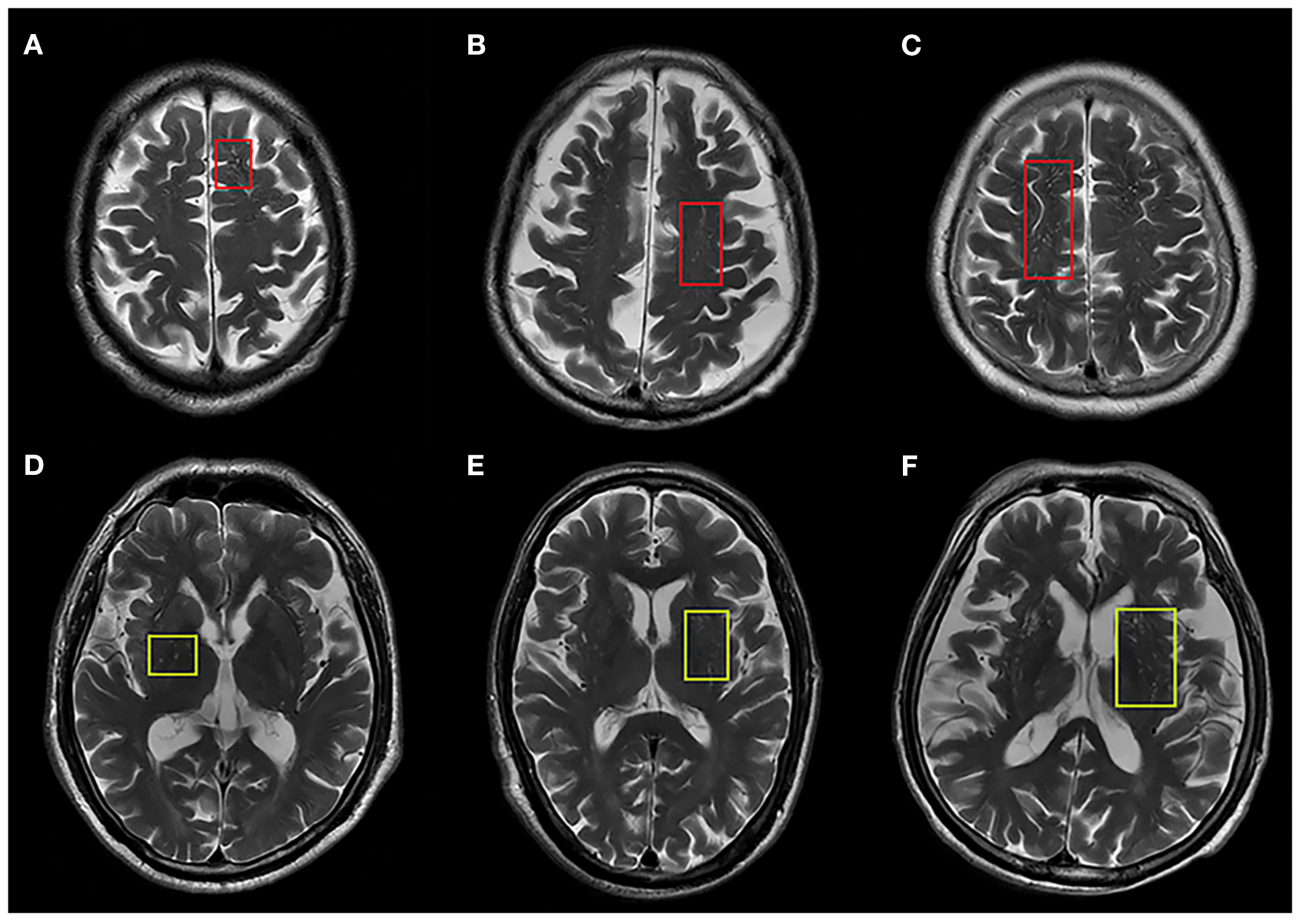
Representative axial T2-weighted images of enlarged PVS in centrum semiovale (ePVS-CSO) and basal ganglia (ePVS-BG). **(A–C)** Indicate ePVS-CSO with red squares corresponding to degree of severity. **(D–F)** Indicate ePVS-BG with yellow squares corresponding to degree of severity.

All patients underwent a cerebral MRI including T1 weighted imaging (T1WI), T2 weighted imaging (T2WI), diffusion weighted imaging (DWI), and fluid attenuated inversion recovery sequence (FLAIR) and T2 star gradient-recalled echo (T2^*^GRE) imaging or susceptibility weighted imaging (SWI). Enlarged PVS and other CSVD imaging makers were rated according to the Standards for Reporting Vascular Changes on Neuroimaging criteria (STRIVE) by two investigators (BY and YC) (17), who were trained by experienced neuroradiologists and blinded to the patients' clinical status. Enlarged PVS were defined as <3 mm round or linear CSF-isointense lesions along the course of penetrating arteries with easily-recognized hyperintensity on T2 images. Lacunes, defined as round or ovoid cavities of 3–15 mm in diameter, were characterized by a hypointense lesion and hyperintense rim on FLAIR images. WMH were defined as hyperintense lesions on FLAIR images without cavitation. A validated 4-point visual rating scale (0, no PVS; 1, 1–10 PVS; 2, 11–20 PVS; 3, 21–40 PVS; 4, >40 PVS) was used for grading PVS in the centrum semiovale (CSO) and basal ganglia (BG) (18). PVS was counted in both hemispheres, and the number of PVS referred to that of hemisphere slice with the highest number of PVS after all the relevant slices for each anatomic area were checked. Severity of PVS was defined as none/mild (rating scale 0–1), moderate (rating scale 2), and severe (rating scale 3–4) (Figure 1). The inter-rater agreement was good for enlarged PVS-CSO [intraclass correlation coefficient (ICC) = 0.69] and PVS-BG (ICC = 0.76), respectively. Additionally, WMH was rated according to the Fazekas scale (19) and the total number of lacunes was also recorded.

### Statistical analysis

Data were expressed as number and percentage for categorical variables, and mean with standard deviation or median with interquartile range for continuous variables. The Chi-squared test was performed for categorical variables, and two-sample *t*-test and Mann-Whitney test were performed for continuous variables in normal and abnormal distribution, respectively. Multivariate logistic regression model was used to analyze the association of enlarged PVS with FOG in patients with PD, after adjustment for the possible confounders and variables with *P* < 0.10 in the univariate analysis. The data with missing outcomes were removed from the analysis. Among PD patients stratified according to the severity of enlarged PVS, Chi-squared test for categorical variables and Kruskal-Wallis test for continuous variables along with Bonferroni correction were applied. Spearman's correlation analysis was conducted to explore the correlation between enlarged PVS number and FOGQ score. P < 0.05 was considered statistically significant. Statistical analysis was conducted with SPSS 24.0 (SPSS Inc, Chicago, IL).

## Results

### Patient demographics and characteristics

Of 126 patients with PD during the study period, a total of 95 patients were included in the final analysis, after excluding patients with contraindications to MRI scan (2 patients), history of stroke (6 patients), an inability to cooperate or communicate (3 paitents), obvious WMH (9 patients), lacunes (6 patients), microbleeds (2 patients), gait problems with possibility of being secondary to orthopedic issues (3 patients). There were 36 PD patients (37.9%) grouped into PD-FOG (+), with an older age and a longer disease duration compared with D-FOG (-) group (75 [71-82] vs. 71[65-77], *p* = 0.008; 7 (5–10) vs. 5 (3–7), *p* = 0.005, respectively). The numbers of enlarged PVS in both CSO and BG were significantly higher in patients with FOG than those without FOG (19.5 [8.3-33.0] vs. 8.0 [5.0-15.0], *p* < 0.001; 8.0 [5.3-16.0] vs. 4.0 [3.0-7.0], *p* < 0.001, respectively). The burden of enlarged PVS based on the severity grading in both regions were different between two groups (*p* = 0.001 and *p* = 0.006, respectively). There was no statistically significant difference between two groups regarding the gender, education level, the prevalence of medical history, H-Y stage, UPDRS-III score, as well as the MMSE and MOCA scores. Neuroimaging manifestations including WMH and lacunes were similar between two groups of patients due to the criteria of our study. The demographics and characteristics of the patients are summarized in Table 1.

**Table 1:** Patient demographics and characteristics between PD patients with FOG and without FOG.

	**PD-FOG (+)**	**PD-FOG (-)**	***P***
	**(*n =* 36)**	**(*n =* 59)**	
Age (years), median (IQR)	75 (71–82)	71(65–77)	**0.008**
Male, no. (%)	21 (58.3)	29 (49.2)	0.385
Education level (years), median (IQR)	7.5 (3.0–10.5)	9.0 (6.0–12.0)	0.287
**Medical history, no. (%)**
Hypertension	20 (55.6)	24 (40.7)	0.158
Diabetes mellitus	10 (27.8)	13 (22.0)	0.526
Hyperlipemia	11 (30.1)	19 (32.2)	0.867
Coronary heart disease	9 (25.0)	16 (27.1)	0.820
Smoking history, no. (%)	10 (27.8)	14 (23.7)	0.659
Drinking history, no. (%)	8 (22.2)	10 (16.9)	0.525
PD disease duration (years), median (IQR)	7 (5–10)	5 (3–7)	**0.005**
H-Y stage, median (IQR)	3 (3–4)	3 (3–4)	0.215
UPDRS-III (OFF)	66.5 (54.3–73.0)	60.0 (46.0–74.0)	0.112
MMSE score, median (IQR)	24.0 (18.3–27.0)	25.0 (21.0–27.0)	0.238
MOCA score, median (IQR)	19.0 (11.5–22.8)	20.0 (16.0–23.0)	0.181
**Neuroimaging**
ePVS-CSO number, median (IQR)	19.5 (8.3–33.0)	8.0 (5.0–15.0)	**0.000**
ePVS-CSO severity, no. (%)			**0.001**
None/mild	11 (30.6)	39 (66.1)	
Moderate	7 (19.4)	8 (13.6)	
Severe	18 (50.0)	12 (20.3)	
ePVS-BG number, median (IQR)	8.0 (5.3–16.0)	4.0 (3.0–7.0)	**0.000**
ePVS-BG severity, no. (%)			**0.006**
None/mild	21 (58.3)	50 (84.7)	
Moderate	9 (25.0)	4 (6.8)	
Severe	6 (16.7)	5 (8.5)	
WMH, median (IQR)	1 (0–1)	1 (0–1)	0.408
Lacunes number, median (IQR)	0 (0–1)	0 (0–0)	0.662

FOGQ, freezing of gait questionnaire; MMSE, Mini-Mental State Examination; MoCA, Montreal Cognitive Assessment; ePVS, enlarged perivascular spaces; CSO, centrum semiovale; BG, basal ganglia; WMH, white matter hyperintensity; IQR, interquartile range; SD, standard deviation.

The bold font indicates that the difference achieved statistical significance (p < 0.05).

### The association of enlarged PVS with FOG in patients with PD

The univariate analysis showed that the age, disease duration, the burden of enlarged PVS based on the severity grading in either region (CSO and BG) were significantly associated with FOG in patients with PD (*p* < 0.05). The multivariate logistic regression analysis further indicated that only the association of the highest-severity degree of enlarged PVS in the region of CSO with FOG remained markedly (adjusted odds ratio [OR], 3.869; 95% confidence interval [CI], 1.213-12.339; *p* = 0.022), independent of factors with a P value < 0.10 in the univariate analysis (age and disease duration) and UPDRS-III score. We found no association between any severity degrees of enlarged PVS located in BG and FOG in PD patients (Table 2).

**Table 2:** Univariate and multivariate logistic regression analysis for the association of enlarged PVS with FOG in PD patients.

**Variables**	**Univariate analysis**		**Multivariate analysis**	
	**OR (95% CI)**	***P***	**OR (95% CI)**	**P**
Age	1.080 (1.019–1.145)	**0.009**	1.024 (0.936–1.121)	0.600
Disease duration	1.178 (1.037–1.338)	**0.012**	1.159 (0.989–1.357)	0.068
UPDRS-III (OFF)	1.025 (0.995–1.056)	0.107	0.995 (0.951–1.042)	0.837
ePVS-CSO severity		**0.003**		**0.048**
None/mild	1(Ref)		1(Ref)	
Moderate	3.102 (0.920–10.458)	0.068	3.305 (0.842–12.967)	0.087
Severe	5.328 (1.975–14.321)	**0.001**	3.869 (1.213–12.339)	**0.022**
ePVS-BG severity		**0.018**		0.543
None/mild	1(Ref)		1(Ref)	
Moderate	5.357 (1.485–19.332)	**0.010**	2.240 (0.492–10.186)	0.297
Severe	2.857 (0.785–10.396)	0.111	1.591 (0.344–7.354)	0.552

OR, odds ratio; CI, confidence interval; Ref, reference; MoCA, Montreal Cognitive Assessment; ePVS, enlarged perivascular spaces; CSO, centrum semiovale; BG, basal ganglia; WMH, white matter hyperintensity.

The bold font indicates that the difference achieved statistical significance (p < 0.05).

### Clinical characteristics of patients with PD according to the severity and location of enlarged PVS

Either region of enlarged PVS was divided into three groups based on severity as described in the Material and methods section. The percentages of FOG case increased accompanied by the aggravation of enlarged PVS located in CSO. There was significantly greater likelihood of FOG in group with severe degree of enlarged PVS-CSO compared to those with none/mild degree (adjusted *p* = 0.001). Meanwhile, the change of percentages of FOG case was significant, but not simultaneous, among different burden groups of enlarged PVS-BG. The age of patients with severe enlarged PVS-CSO burden were significantly higher than those with none/mild burden. Patients with increasing burden of enlarged PVS-BG have older age (*p* = 0.009), but the significance disappeared after post hoc analysis. Patients with severe degree of enlarged PVS-BG had lower MMSE scores than those with none/mild degree (adjusted *p* = 0.012). There were no statistically significant differences among the groups regarding other characteristics (Table 3).

**Table 3:** Clinical characteristics of patients with PD according to the severity and location of enlarged PVS.

	**ePVS-CSO**				**ePVS-BG**			
	**none/mild**	**Moderate**	**Severe**	***P***	**None/mild**	**Moderate**	**Severe**	***P***
	**(*n =* 50)**	**(n =15)**	**(*n =* 30)**		**(*n =* 71)**	**(*n =* 13)**	**(*n =* 11)**	
Age	70 (66.5–75)	78 (71–83)	75 (70–81)^a^	**0.026**	71 (65–78)	73 (71–83)	77 (72–85)	**0.009**
Disease duration (years)	5.0 (3.0–7.5)	5 (3–8)	6 (4.5–10)	0.304	5 (3–7)	8 (3–10)	5 (3–10)	0.084
H-Y stage	3 (3–4)	3 (3–4)	3 (3–4)	0.861	3 (3–4)	3 (3–3.5)	4 (3–4)	0.228
UPDRS-III (OFF)	61.5 (48–73)	63 (46–80)	62.5 (54–74)	0.767	59 (48–72)	67 (62–69)	74 (59–80)	0.057
FOG case, no. (%)	11 (22.0)	7 (46.7)	18 (60.0)^b^	**0.002**	21 (29.6)	9 (69.2)^c^	6 (54.5)	**0.012**
MMSE score	25 (21–27)	23 (20–26)	24.5 (19–28.5)	0.387	25 (21–27)	23 (20.5–28)	19 (18–22)^d^	**0.019**
MOCA score	20 (16–23)	19 (14–22)	21 (12–26)	0.556	21 (16–23)	19 (13.5–24)	13 (7–20)	0.063

^a^Patients with severe degree of ePVS-CSO had older age than those with none/mild degree of ePVS-CSO (adjusted p = 0.042).

^b^Patients with severe degree of ePVS-CSO had higher percentage of FOG case than those with none/mild degree of ePVS-CSO (adjusted p = 0.001).

^c^Patients with moderate degree of ePVS-BG had higher percentage of FOG case than those with none/mild degree of ePVS-BG (adjusted p = 0.006).

^d^Patients with severe degree of ePVS-BG had lower MMSE scores than those with none/mild degree of ePVS-BG (adjusted p = 0.012).

ePVS, enlarged perivascular spaces;MMSE, Mini-Mental State Examination; MoCA, Montreal Cognitive Assessment; CSO, centrum semiovale; BG, basal ganglia.

The bold font indicates that the difference achieved statistical significance (p < 0.05).

### Correlation analysis of enlarged PVS with FOGQ score

**Figure 2:**
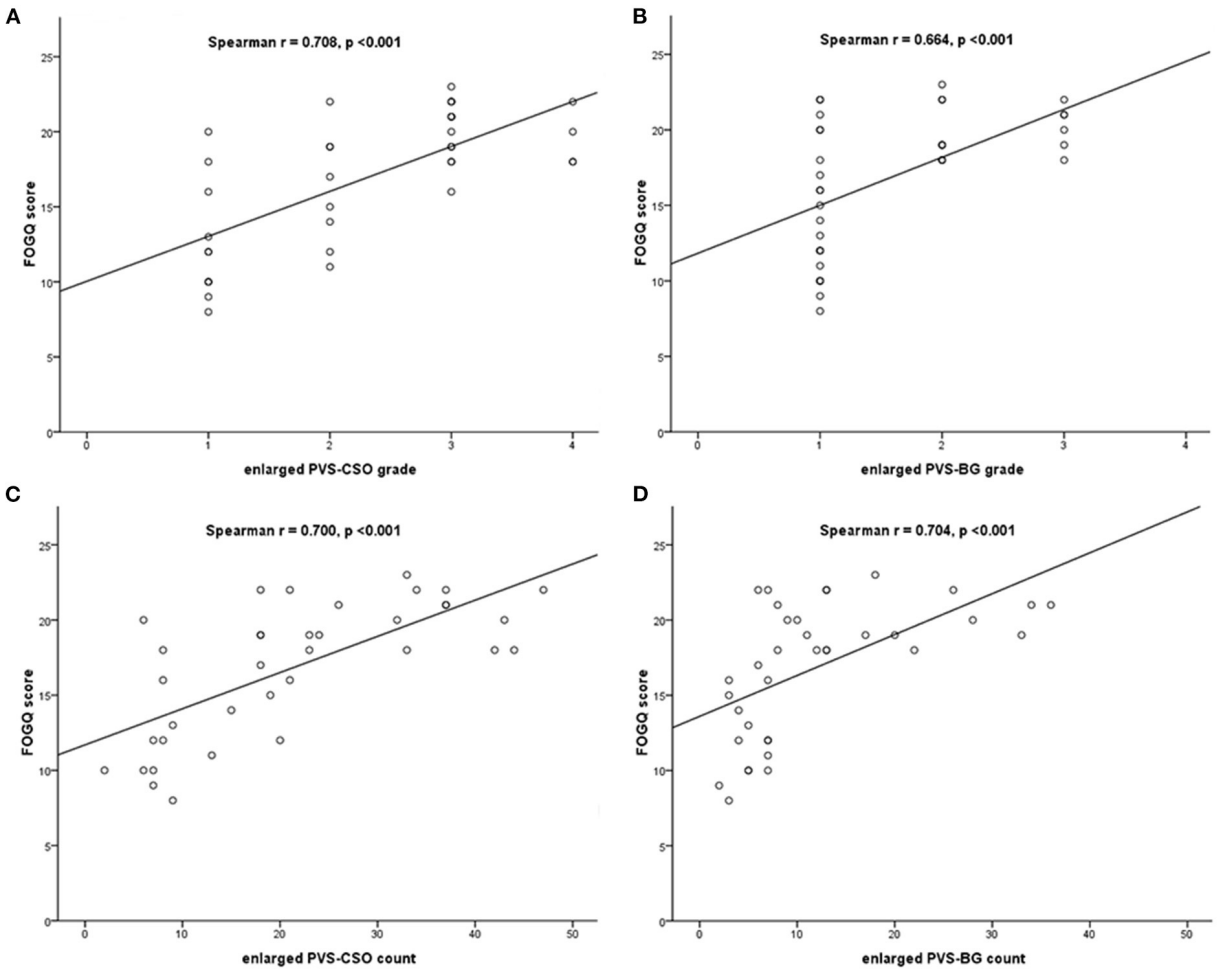
Spearman's correlation between the enlarged PVS and FOGQ score in PD patients with FOG. **(A)** Correlation between enlarged PVS-CSO grade and FOGQ score. **(B)** Correlation between enlarged PVS-BG grade and FOGQ score. **(C)** Correlation between enlarged PVS-CSO count and FOGQ score. **(D)** Correlation between enlarged PVS-BG count and FOGQ score.

Spearman's correlation analysis adjusted for covariates including age, disease duration, MDS-UPDRS III score, MMSE and MoCA score, demonstrated that the grade of enlarged PVS in CSO and BG using the 4-point rating scale were significantly correlated with FOGQ score (Spearman *r* = 0.708, *p* < 0.001; Spearman *r* = 0.664, *p* < 0.001, respectively), and the count of enlarged PVS in both regions were also correlated with FOGQ score (Spearman *r* = 0.700, *p* < 0.001; Spearman *r* = 0.704, *p* < 0.001, respectively) in PD patients with FOG (Figure 2).

## Discussion

To the best of our knowledge, the present study is the first to report the association of enlarged PVS with FOG in patients with PD in the absence of extensive WMH and lacunes, avoiding the potential confounding effect of common CSVD imaging markers. Our results indicated that PD patients with FOG had significantly higher numbers of enlarged PVS distributed in both CSO and BG compared with those without FOG. Enlarged PVS burden in CSO and BG were positively correlated with the FOGQ score in PD patients with FOG. Furthermore, enlarged PVS in the region of CSO with the highest-severity degree was independently associated with FOG.

A number of factors have been identified to be associated with FOG in PD patients, including disease duration, motor phenotypes, CSVD neuroimaging markers, cerebrospinal fluid β-amyloid 42 (Aβ42), dopamine transporter (DAT) uptake, and cognitive impairment (20–22). However, the studies exploring the role of PVS in the FOG of PD are still scarce. A recent research by Lv et al. (14) applying high-resolution 7T-MRI focused on the association of normal-sized PVS with FOG in PD patients. Similar to our study, Lv el al. found that the number of normal-sized PVS in BG was significantly higher in PD patients with FOG than those without FOG, while no significant difference in number of normal-sized PVS in CSO was detected among the participants including healthy controls, contrary to our result. Nevertheless, it was noted that the semi-quantitative assessment of normal-sized PVS severity in Lv's study indicated that FOG group had a more severe PVS burden compared with non-FOG group in PD patients, which was partially consistent with our result. The conflicting reports between the two studies might be explained by the potential pathophysiological differences between normal-sized and enlarged PVS, as well as the limitation of relatively small sample size and lack of logistic regression analysis in the Lv's study. Another previous study (23) observed that enlarged PVS score in CSO was higher in PD patients of PIGD motor phenotype than that in non-PIGD motor phenotype, while enlarged PVS score in BG had no significant difference in two groups. Furthermore, enlarged PVS in CSO indicated a tendency toward a significant difference in the logistic regression analysis of PIGD phenotype. Although PD patients with FOG were not singled out from the PIGD phenotype, this study hinted that enlarged PVS in CSO might play a role in gait function in PD as our study. Meanwhile, a positive correlation between enlarged PVS in BG and the tremor score was displayed, which was just opposite to the finding from Lv et al. that a negative correlation existed between normal-sized PVS count in BG and the tremor score. Additionally, a recent study showed that enlarged PVS in BG had no relation with gait/postural instability but only correlated with cognitive impairments in PD patients (24), the latter finding of which was also indicated in our study. Notably, this study did not mention the assessing of PVS in CSO and also did not group the FOG patients. The heterogenicity in selected participants of those studies may explain the contradiction of results concerning the link of PVS with different PD motor phenotypes. What are mentioned above give us a clue that the association between PVS and gait impairment was still controversial, and the effect of enlarged PVS on FOG in PD patients could not be ruled out. Therefore, additional studies are needed for further exploration and confirmation.

Meanwhile, older age and longer disease duration were found in FOG group compared with non-FOG group in our study, however, they failed to show significant association with FOG in multivariate logistic regression analysis, contrary to prior evidence (20, 25). In addition, in conflict with the PPMI study indicating that lower MoCA score was a significant predictor of FOG in PD (21), no link between cognition and FOG was found in our study, which may be partially attributable to exclusion of PD patents with extensive WMH or lacunes, given that WMH and lacunes have been reported to be associated with cognitive decline in PD patients (5, 26).

By analyzing the data based on the severity and location of enlarged PVS, we found that PD patients with more severe burden of enlarged PVS in CSO were more prone to have FOG, whereas patients with more severe burden of enlarged PVS in BG were more likely to display impaired cognition. The intriguing results hinted the different roles of PVS in different regions in PD patients. Similarly, studies from Park et al. (27) and Chen et al. (28) discovered that higher severity of PVS in BG predicted cognitive decline in PD patients. Nevertheless, Chung et al. (29) found that PD patients with more enlarged PVS in BG had more risk of developing of FOG, in contrast to our study. Therefore, future studies are required to elucidate the mystery.

An innovation of the present study was that PD patients with extensive WMH and lacunes that may contribute to the gait impairment were not included. It has been reported that PD patients with moderate to severe WMH displayed a higher risk of developing FOG than those with mild WMH (30), and lacunes especially in BG might independently lead to gait/posture dysfunction in PD (31). Moreover, in PD patients, severer WMH was shown to be linked to more cognitive deficits in the domains potentially affecting gait performance such as attention and executive function (26). We therefore speculated that it would be much difficult to separate WMH-induced gait impairment from FOG due to PD per se when encountering a PD patient with apparent WMH or lacunes in clinical scenarios. In order to better explore the contribution of enlarged PVS on FOG in the “pure PD” population, we tried to exclude PD patients with common vascular and other potential influencing factors of gait in our study, which to a great extent weakened the heterogeneity of participants and avoided the confounding effect of these factors on FOG.

The underlying pathophysiology leading to FOG in PD patients remains unclear. Based on relevant publications, we try to conjecture it from the perspective of PVS. First, gait function closely depends on the elaborate coordination of the specific brain regions, such as mesencephalic locomotor region (MLR), pontomedullary reticular formation (PMRF), basal ganglia, cerebral cortex, and cerebellum (32). Also, the complex and intact interaction of these regions with the whole neural network is indispensable for normal gait (2). Severe enlarged PVS might lead to impaired cerebrovascular reactivity, blood-brain barrier dysfunction, and perivascular inflammation (33). Although the region of CSO seems not to be essential to gait function as BG, we infer that enlarge PVS in CSO are likely to interrupt the cortico-basal ganglia/thalamus and cortico-cortical connectivity, and lead to FOG in PD. Second, there has been evidence from an *in vivo* positron emission tomography (PET) study indicating that extra-nigral pathologies, including cortical Aβ deposition and cholinergic denervation, contribute to FOG (34). Kim et al. discovered that CSF Aβ42 was associated with the development of FOG in PD (20). In view of the notion that enlarged PVS in the cortex are likely to accumulate Aβ, distinct from the PVS in BG (35, 36), we therefore speculated that enlarged PVS in CSO might indirectly contribute to FOG through the mediation of Aβ. Third, enlarged PVS reflect the poor interstitial fluid drainage that could diminish clearance of α-synuclein and subsequently accelerate the progression of FOG.

Several limitations of our study also should be noted. First, it was a cross-sectional and single-center study, thus the causality between enlarged PVS and FOG cannot be identified, and the sample population may fail to represent the general population. Future longitudinal studies with multicenter data and larger sample size are needed to further explore the role of enlarged PVS in FOG. Second, although FOGQ is a widely applied rating scale to define FOG and assess FOG severity, it cannot precisely quantify the FOG characteristics. Emerging technology-assisted devices of gait analysis are expected to provide objective and quantitative data on FOG gait parameters in PD patients (37), thus to assist in clarifying the links of PVS and FOG.

In conclusion, the present study demonstrates that enlarged PVS, particularly in CSO, are associated with FOG in patients with PD. Our findings could provide a novel explanation for pathophysiological mechanisms of FOG in PD. Further prospective longitudinal studies are expected to confirm our observation.

## Data availability statement

The raw data supporting the conclusions of this article will be made available by the authors, without undue reservation.

## Ethics statement

The studies involving human participants were reviewed and approved by the Ethics Committee of Beijing Shijingshan Hospital, Shijingshan Teaching Hospital of Capital Medical University. The patients/participants provided their written informed consent to participate in this study.

## Author contributions

WJ and FL contributed to the conception and design of the study, statistical analysis, and drafting the manuscript. WZ and BL contributed to the recruitment of subjects and collecting clinical data. BY and YC contributed to the assessment of neuroimaging. WJ contributed to the study supervision. All authors contributed to the article and approved the submitted version.

## Funding

The study was supported by the Capital Health Research and Development of Special Program (2020-3-7091).

## Conflict of interest

The authors declare that the research was conducted in the absence of any commercial or financial relationships that could be construed as a potential conflict of interest.

## Publisher's note

All claims expressed in this article are solely those of the authors and do not necessarily represent those of their affiliated organizations, or those of the publisher, the editors and the reviewers. Any product that may be evaluated in this article, or claim that may be made by its manufacturer, is not guaranteed or endorsed by the publisher.
